# Nature-Based Interventions and Exposure among Cancer Survivors: A Scoping Review

**DOI:** 10.3390/ijerph20032376

**Published:** 2023-01-29

**Authors:** Erica R. Timko Olson, Anthony A. Olson, Megan Driscoll, Amber L. Vermeesch

**Affiliations:** 1 School of Nursing, University of Minnesota—Twin Cities, 308 Harvard St. SE, Minneapolis, MN 55455, USA; 2 St. John’s University, Collegeville, MN 56321, USA; 3 Department of Family and Community Nursing, School of Nursing, University of North Carolina Greensboro, Greensboro, NC 27403, USA

**Keywords:** cancer, neoplasms, nature-based therapy, forest therapy, survivorship

## Abstract

Background and purpose: nature-based interventions (NBI) have been shown to have positive effects on physical, psychological, social, and spiritual health. The purpose of this scoping literature review was to describe what is known regarding the cancer survivor experience in relationship to their interaction with the natural environment. Description/methods: this review was completed using the Preferred Reporting Items for Systematic reviews and Meta-Analyses extension for Scoping Reviews (PRISMA-ScR). The research strategy included a combination of these terms: cancer, neoplasms, nature, and forest therapy. The articles were blinded and screened by four independent researchers. A total of twelve articles were selected. Outcome/results: a total of 2786 cancer survivors participated in the twelve studies with multiple types and stages of cancer represented. The studies used multiple designs and measures. Results showed improvements in anxiety, depression, sleep, connectedness, stress, tension, confusion, fatigue, and pain. Participants reported that nature was the most important resource in coping with their cancer. Conclusions and implications: nature is beneficial for cancer survivors while they experience cancer diagnosis and treatment. Nature opportunities can be feasibly delivered with this population and need to be explored further and safely implemented to support the overall health and well-being of cancer survivors.

## 1. Introduction

Cancer is the leading cause of death worldwide with almost 10 million deaths occurring in 2020. The most common new cases are due to breast, lung, and colon cancer, but cervical cancer is the most common in 23 countries. Cancer death is best reduced by early detection and treatment [1]. Childhood cancer rates have been steadily increasing worldwide during the last forty years, but mortality rates have declined significantly [2]. More children and adults are surviving cancer than ever before. This increase also comes with additional burdens of the long-term negative mental and physical effects of cancer. The burden of cancer survivorship is stressful, with 25% of survivors reporting problems such as anxiety, depression, and psychological as well as psychosocial distress. Perceived stress is associated with higher symptom burden for cancer survivors [3]. For rural cancer survivors, financial burdens related to their cancer are higher than for urban cancer survivors, thus impacting cancer survivorship burden for rural individuals [4]. These factors can cause additional suffering and affect adherence to treatments and the survivors’ overall health and well-being [5]. 

Nature-based interventions (NBI) have continued to show promising effects on overall health and well-being in the published research over the last forty years. There continues to be more attention given to the positive effects of NBI on physiological as well as psychological improvements. The psychological benefits of NBI are significant and can play a role in both prevention and treatment of mental health disorders [6]. NBI also promotes overall psychological well-being and can reduce the burden of anxiety, depression, and stress [7].

There is a dearth in the existing literature regarding the impact on cancer survivorship of spending time in nature, despite the known impact on improved wellness and well-being for vulnerable populations. We were unable to find an existing scoping review examining NBIs and exposure among cancer survivors published within the last ten years. There are examples of systematic reviews and qualitative meta-synthesis regarding cancer-related outcomes [8] and the role of nature in cancer patients’ lives [9] but not on cancer survivorship. NBIs are effective interventions to reduce anxiety, depression, and psychological as well as psychosocial distress among individuals diagnosed with cancer [9,10,11,12,13,14,15,16,17] and should be utilized to promote well-being among cancer survivors.

In the available systematic reviews, NBIs are shown to have positive impacts on cancer-related quality of life, physical activity, immune system markers such as natural killer cells and T cells, cortisol levels, anxiety levels, and blood pressure [8]. Cancer survivors have many closely linked common risk factors for cardiovascular disease including increased age, poor diet, psychosocial stress, inflammation, and sedentary lifestyles that are positively impacted by NBIs [18]. Inflammation is a key symptom that has shown to be improved by NBIs and is particularly important among cancer survivors [18]. 

Despite its efficacy in improving overall well-being, there is a lack of existing consensus on the definition of nature and, specifically, of NBIs, but three conditions have generally been considered: viewing nature, being surrounded by nature, and active involvement with nature [19,20]. Definitions range from programs, activities, or strategies aimed at engaging people in nature-based experiences with specific well-being goals [21] to forest bathing, nature viewing, nature visits, and park visits [8]. NBIs are also referred to as green care, ecotherapy, forest therapy, and nature-based therapy [19]. Other definitions define nature exposure by objective measures accompanied by their own confounding variables (i.e., greenspace and proximity, wooded areas, and access) [22]. Current literature defines nature exposure using such metrics as existence and amount of greenspace, but this is limited as it does not encompass access to, quality of, or usability of greenspace [22]. Generally, NBIs are considered planned, intentional activities to promote aspects of well-being occurring in natural environments. According to Gritzka 2020, NBIs are effective because of the evolutionary predisposition of humans to pay attention to natural environments [23]. 

To improve understanding of the multiple burdens of cancer survivorship and the known benefits of NBIs, a scoping review of the literature was conducted on the relationship of NBI and exposure among cancer survivors.

The purpose of this scoping review was to systematically describe the current knowledge about the role of nature in the cancer survivor experience from diagnosis through the end of life. The following research question was created: What is currently known from the literature regarding the cancer survivor experience in relationship with their interaction with a natural environment, viewing nature, being surrounded by nature, and/or active involvement with nature?

## 2. Materials and Methods

The methods for the scoping review were designed following the Preferred Reporting Items for Systematic reviews and Meta-Analyses extension for Scoping Reviews (PRISMA-ScR) (Table 1), which was developed by Tricco et al. [24] according to guidelines by the EQUATOR (Enhancing the QUAlity and Transparency Of health Research) Network. The review protocol was not registered.

For an article to be considered for this scoping review, it was required to meet the following inclusion criteria. The primary research article must have been published between 2013 and 2022, as the previous review was completed ten years ago, available in English, full-text, published in a peer-reviewed journal, and involve human participants. The literature search included all cancer survivor populations from pediatric to older adult that included a nature-based intervention, a focus on the nature environment for the intervention, or a virtual-reality experience in nature. Surveys were also included when they described the cancer survivors’ experience and/or relationship with nature. Pilot studies, secondary analysis, qualitative, quantitative, and mixed-methods research were all included to gain a comprehensive understanding of the current literature. A comparison group was not required. Research studies that focused on the evaluation of greenspace as it relates to cancer incidence and rates were excluded from this review due to the focus on epidemiology rather than on relationships and interactions with nature.

The following databases were searched from 2013 through October 2022: MEDLINE, Alt Health Watch, PubMed, ProQuest, and Cochrane. The search strategy was devised in consultation with a medical research librarian and included iterations of the following terms individually and in combination: cancer, neoplasms, survivor, nature, nature-assisted therapy, nature-based intervention, forest bathing, forest therapy, blue space, greenspace, health, and well-being. 

Cancer survivors are defined as those from diagnosis through death, at any stage of the disease process. The terms for nature are varied and encompass terms such as greenspace, forest, nature bathing, and forest bathing, with the focus on interaction and relationship within these spaces. The landscape may be green (trees, plants, grasses) or blue (ocean, lake, river, stream).

**Figure 1:**
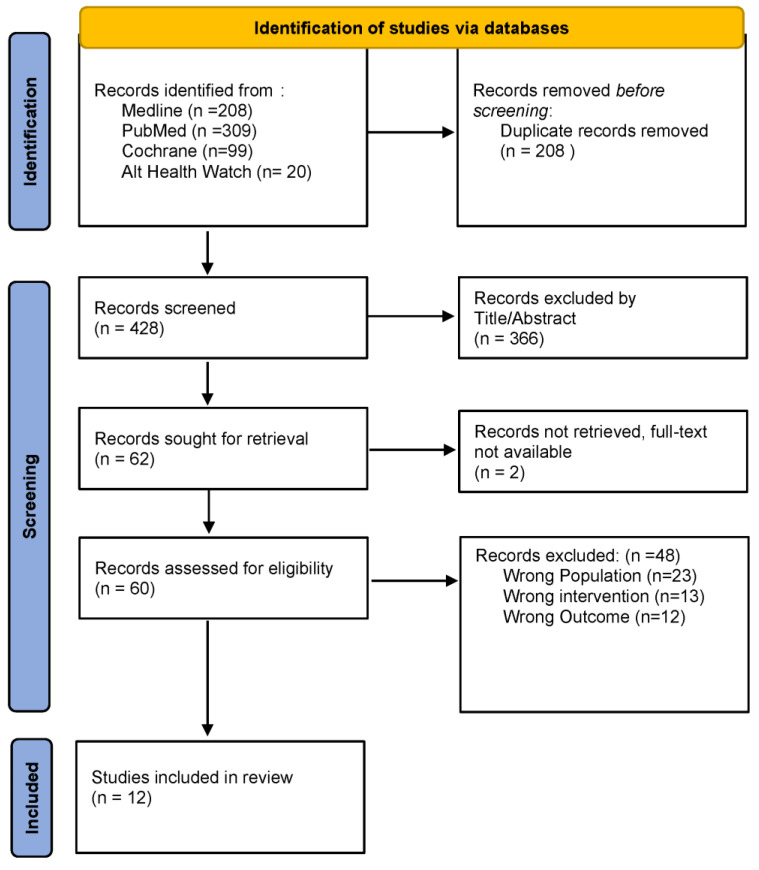
Flow diagram [26].

A total of 636 records were identified through database searches and populated in RAYYAN, an online data management web-based resource that allows for online collaboration with researchers around the world [25]. After removal of 208 records that were duplicates, 428 records were screened by four independent reviewers. An additional 366 records were removed after the review of the title and/or abstract. The screening involved classification of articles as ‘include’, ‘maybe’, or ‘exclude’ and the rationale for exclusion, such as wrong publication, wrong language, wrong outcome, or wrong population. If there was disagreement on inclusion or exclusion, the four reviewers discussed the article and came to a consensus decision. A total of 62 records were sought for retrieval. An additional 2 records were not available in full-text format, resulting in 60 full-text records. Four reviewers independently evaluated the full text of the selected records for potentially relevant publications with discussion and consensus. Twelve studies were included in the final review; the process is summarized in a flow diagram (Figure 1). Data extraction was performed by two independent reviewers, and data from each reviewer were collated, discussed, summarized, and synthesized (Table 2). Data were extra on author, year, country of origin, research question, aims, or purpose; participants; intervention; instruments; and results. This charting form has been used previously by the research team.

## 3. Results

Table 2 is a summary of the selected articles and the individual characteristics. Studies were published between 2013 and 2022, with nine (75%) published in 2018 or later. 

### Study Characteristics

The total number of participants in the twelve studies [11,12,13,14,15,16,17,27,28,29,30,31] reviewed was 2786. The study with the smallest sample had 9 participants [29]. The largest sample had 2417 [17] participants, with the next largest having 80 participants [28]. The mean number of participants, excluding the outlier, was 33.63, and the mode was 30. The ages of the participants ranged from 18 to 85 years with a mean age of 50.4 years. Most of the studies were conducted in the United States [12,13,31] (3), followed by Australia [15,27] (2), Sweden [17] (1), the Netherlands [16] (1), New Zealand [11] (1), Iran [28] (1), Korea [29] (1), Canada [14] (1), and Japan [30] (1). The number of male participants was 530 (19%), and the number of female participants was 2256 (81%). When excluding the outlier study, there were 6% males and 94% females. Nine of the studies included both males and females, and the remaining three focused exclusively on females with breast cancer. Data on race and ethnicity were not reported in the studies. The most common types of cancer reported were breast (8), blood (4), GI (3), gynecological (2), brain (2), bone (2), lung (2), urology (1), and skin (1). 

Regarding study design, one was secondary data analysis (8%) of a randomized control trial, [11] two were observational surveys (16.7%), [13,17] two used a mixed-methods design (16.7%) [14,16], and two had qualitative designs (16.7%) [15,27]. The remaining five studies were quantitative studies (42%) using descriptive correlational [28,29,30], repeated measures [12], and program evaluation [31]. Two of the studies stated they were pilot studies [16,30], and one was identified as an exploratory study [29].

The aim statements of the studies can be summarized into two main categories. The researchers designed studies to explore and exchange cancer patients’ experiences and the role that nature had in their coping and identity as a cancer survivor. The second category was the clear focus on designing effective and sustainable nature-based interventions to support the cancer survivor experience.

Eight of the studies (66%) were interventional studies. Two of the studies involved week-long retreats in a forested or natural environment [16,29], and one involved either a week-long or weekend experience [31]. Two of the studies involved virtual-reality nature experiences, either daily or during a painful procedure [11,12]. One study compared viewing nature or the city from a hospital window [28], while the remaining studies involved a nature-rich exercise program for 10 weeks [14] and spiritual care (forest therapy and horticulture therapy) in an urban green space [30].

The studies used standardized and investigator-developed questionnaires for qualitative and quantitative data collection (Table 3). The studies collected data regarding the association between nature interventions and anxiety [11,28,29,30,31], stress [11,12,13], depression [11,29,31], pain [11,12,28], fatigue [11,14,30], sleep [29,31], quality of life [11,30], nature relatedness [13,14], and spiritual well-being [11,30] outcomes. The studies also examined general well-being [16], cancer identity [16], nature connectedness [11], a symptom inventory [13], psychological state [30], function [14], inflammatory biomarkers [31], and natural killer cell activity [30]. 

The findings can be summarized into three distinct categories. Indirect engagement involves viewing nature, listening to nature sounds, or experiencing nature through virtual reality. Incidental engagement refers to walking, resting, or relaxing in the outdoors. Intentional engagement with nature refers to adventure therapy, forest therapy, and intentional immersive time and experiences in nature.

Indirect engagement with nature has been shown to help with relaxation, improve sleep, help people generally feel better, and provide comfort [15,17,27]. It can be used to create virtual distraction and offer engaging visualization that can reduce high symptom distress, pain, tension, and state–trait anxiety [13,27,28,30].

Incidental engagement with nature resulted in less pain and state–trait anxiety, improved mood, and was beneficial for self-reflection [14,16,17,28]. It supported well-being, QOL, relaxation, improved fatigue, and facilitated social interaction [14,15,16,30]. The body relaxes when surrounded by nature, and it supports stillness, quietness, and relatedness, reduces fear, lowers stress, and increases natural killer cell activity [13,14,15]. Physical activity engagement in nature is enhanced, and aerobic fitness improves, but functional limitations can also be enhanced. Nature enables a shift in the mind and seems effortless, as nature does not recognize cancer, so people can simply relax and enjoy nature, lose themselves, and release control [14,15,27,30]. 

Intentional engagement in nature can be calming, centering, and rejuvenating [12,15]. It can improve fatigue, depression, QOL, sleep, and bring awareness to a new perspective by invoking feelings of awe, joy, and appreciation for the simple things [7,9,11,15,16]. Intentional engagement can increase connectedness to nature, the outdoors, as well as other survivors and support insight into causes of stress, frustration, or discomfort [31]. 

## 4. Discussion

This review examined what is currently known regarding the cancer survivor experience in relationship to their interaction with a natural environment. Twelve studies were identified that addressed nature-based intervention with 2786 cancer survivors, across multiple settings, published between 2013 and 2022. Cancer remains the leading cause of death worldwide, while at the same time more children and adults than ever are surviving cancer. With this survivorship comes many burdens that include stress, anxiety, depression, as well as financial and psychosocial concerns. Nature-based interventions have shown promising effects on overall health and well-being in the general population. Research on the benefits of nature engagement is continually increasing, with many positive outcomes. Additionally, specific research on cancer survivor engagement with nature has increased in the last ten years. Previous reviews have focused on qualitative studies [7,20] and greenspace and cardiovascular health and cancer-related outcomes [6]. This review builds on previous knowledge by including research on additional quantitative as well as qualitative studies and expanding NBIs from greenspace (gardening and forest bathing) to include nature VR, viewing landscapes, exercise in nature, forest therapy, and nature treks. Blaschke [7] reported that nature was a source of strength and meaning that allowed unburdened and uninterrupted space to address their needs related to cancer. Previous work has supported benefits to fatigue, anxiety, and a sense of belonging and self-esteem [20]. In this review, we expanded the scope to examine specific nature interventions that enhance psychological and psychosocial health. Cancer patients and cancer survivors are in a high-stress state, and coping with this stressful experience poses significant challenges [32]. Nature was identified as the most important resource for coping with cancer and promoted self-reflection and relaxation [12,16,17]. Additionally, nature provided a supportive structure to explore the cancer experience and examine the reality of the situation while supporting a deeper connection to self and peers, promoting both self-reflection and social connection [27,31].

This nature engagement through indirect, incidental, and intentional means has been shown to have a positive effect in all the studies reviewed. Nature was beneficial for relaxation, self-reflection, coping, and comfort and supported less fatigue, stress, anxiety, and depression as well as supporting a greater quality of life and spirituality. This is evidenced by the Stress Reduction Theory that describes the therapeutic and restorative effects of nature and natural environments that support recovery from stressful events [32]. The overall benefits to quality of life and well-being are also consistent through the work of McMahan [33], who concluded that there is a wealth of convincing evidence that contact with natural environments supports subjective well-being. 

These findings indicate a variety of methods, interventions, assessment tools, and diagnoses across the cancer survivor population. We found many variables measured using qualitative, quantitative, mixed-methods, and secondary data analysis, with all showing positive results These results are also consistent with the literature across other populations; however, there are several specific gaps in the literature that need to be addressed in future research related to: (1) use of similar and consistent assessment tools; (2) use of standardized interventions (duration, dose, frequency, location descriptions of nature); (3) specific age groups delineated (pediatric, adolescents and young adults, adults, older adults); and (4) specific types and stages of cancer as well as treatment regime (blood, breast, lung, etc.). Research questions should also focus on accessibility and barriers to natural environments as well as motivation to participate in nature-based experiences as cancer survivors. 

## 5. Limitations

This scoping review has some limitations. The review examined only the last ten years of published research in journals that were available only in English. Also, many of the studies had small sample sizes with bias in sampling, with only one randomized controlled trial with a secondary data analysis for this review and two additional studies with a control group. The study protocol was not eligible to be registered with PROSPERO.

## 6. Conclusions

The purpose of this scoping review was to explore what is currently known from the literature regarding the cancer survivor experience in relationship to their interactions with and effects of a natural environment. Cancer survivors interact with nature through indirect, incidental, and intentional ways. The cancer survivor experience is unique yet has many common qualities that nature can support through a variety of interactions. NBIs have good efficacy in mitigating the negative consequences and sequela of cancer treatment and survivorship. Whether viewing nature from a hospital window, experiencing nature VR, walking slowly through a park, or participating in a forest retreat, nature offers many benefits across multiple ages and diagnoses. Previous reviews [8,9,20] have identified the benefits of outdoor physical activity, increased control, and a supportive social environment, and this review expands on previous research by exploring the types of interactions and responses in both quantitative and qualitative studies. This review supports the benefits of NBIs and programs to improve the cancer survivor experience and enhance their well-being. 

## Figures and Tables

**Table 1:** PRISMA extension for Scoping reviews (PRISMA-ScR): checklist [19].

Section	Item	PRISMA-ScR Checklist Item	Page
Title	1	Identify the report as a scoping review.	Title
Abstract			
Structured summary	2	Provide a structured summary that includes (as applicable) background, objectives, eligibility criteria, sources of evidence, charting methods, results, and conclusions that relate to the review questions and objectives.	Title page
Introduction			
Rationale	3	Describe the rationale for the review in the context of what is already known. Explain why the review questions/objectives lend themselves to a scoping review approach.	1
Objectives	4	Provide an explicit statement of the questions and objectives being addressed with reference to their key elements (e.g., population or participants, concepts, and context) or other relevant key elements used to conceptualize the review questions and/or objectives.	2
Methods			
Protocol and registration	5	Indicate whether a review protocol exists; state if and where it can be accessed (e.g., a Web address); and if available, provide registration information, including the registration number.	2
Eligibility criteria	6	Specify characteristics of the sources of evidence used as eligibility criteria (e.g., years considered, language, and publication status), and provide a rationale.	2
Information sources	7	Describe all information sources in the search (e.g., databases with dates of coverage and contact with authors to identify additional sources), as well as the date the most recent search was executed.	2
Search	8	Present the full electronic search strategy for at least one database, including any limits used, such that it could be repeated.	2
Selection of sources of evidence	9	State the process for selecting sources of evidence (i.e., screening and eligibility) included in the scoping review.	3
Data charting process	10	Describe the methods of charting data from the included sources of evidence (e.g., calibrated forms or forms that have been tested by the team before their use, and whether data charting was done independently or in duplicate) and any processes for obtaining and confirming data from investigators.	3
Data items	11	List and define all variables for which data were sought and any assumptions and simplifications made.	2
Synthesis of results	12	Describe the methods of handling and summarizing the data that were charted.	3
Results			
Selection of sources of evidence	13	Give numbers of sources of evidence screened, assessed for eligibility, and included in the review, with reasons for exclusions at each stage, ideally using a flow diagram.	2 Figure 1
Characteristics of sources of evidence	14	For each source of evidence, present characteristics for which data were charted and provide the citations.	3
Results of individual sources of evidence	15	For each included source of evidence, present the relevant data that were charted that relate to the review questions and objectives.	3–4
Synthesis of results	16	Summarize and/or present the charting results as they relate to the review questions and objectives.	4
Discussion			
Summary of evidence	17	Summarize the main results (including an overview of concepts, themes, and types of evidence available), link to the review questions and objectives, and consider the relevance to key groups.	5
Limitations	18	Discuss the limitations of the scoping review process.	5
Conclusions	19	Provide a general interpretation of the results with respect to the review questions and objectives, as well as potential implications and/or next steps.	5
Funding	20	Describe sources of funding for the included sources of evidence, as well as sources of funding for the scoping review. Describe the role of the funders of the scoping review.	5

**Table 2:** Review articles.

Author, Year, Country	Study Design	Research Purpose	Sample Size	Intervention/Methods	Data-Collection Tools	Main Results	Types of Cancer
Ahmadi et al. (2015). [17] Sweden	Survey	Confirm previous results concerning the role of nature as the most important coping strategy among cancer patients in Sweden.	2417	Survey via mail.	Researcher-designed survey.	Nature was the most important resource, followed by listening to the music of nature, and walking or engaging in activities outdoors gave a spiritual sense.	Breast, blood, GI
Albers, T. et al. (2021). [16] Netherlands	Mixed-methods, exploratory, pilot study	AIMS: 1. Create an opportunity for AYA survivors to exchange experiences, foster understanding and recognition, 2. Offer an intervention that stimulates participants to collectively orient themselves on their post-cancer identity and role in society, and 3. Make a first step in the development of an effective and sustainable positive psychology intervention in nature for AYAs after their medical treatment.	13	One week: arrival, departure, and six days of intervention.	Baseline questionnaire focused on: demographics, personal health data, needs and expectations, well-being and cancer identity, post-experience questionnaire, and one-month follow-up.	Nature was beneficial for self-reflection and relaxation.	Breast, blood, gyn, brain, bone
Blaschke, S. et al. (2016). [17] Australia	Qualitative—Grounded Theory	To explore cancer patients’ subjective experiences with nature to examine the relevance of nature-based care opportunities in cancer care contexts.	21	Qualitative.	Convenience sample, then theoretical, semi-structured interview, single interview (mean duration = 54 min).	Nature was a support structure, engagement and survival needed to explore the consequences of cancer, dynamic relationship of cancer’s reality, comfort and safety are necessary.	Blood, bone and soft tissue, GI, gyn, lung, skin, urology, breast
Blaschke, S. et al. (2018). [27] Australia	Qualitative	Gain insight for delineating relevant and translatable nature-based care and design opportunities in oncology contexts.	22	Phone or in-person interviews (mean = 54 min).	Purposive sampling, audio recording, transcribed.	Twelve distinct recommendations for nature engagement emerged: views of nature, desired levels of engagement, and promoting physical activity.	Blood, bone and soft tissue, GI, gyn, skin, breast, urology, lung
Chin, S. et al. (2022). [11] New Zealand	Secondary analysis of an RCT	Aims: explore whether VR nature experiences are associated with physical and psychological benefits for women with MBC who are disconnected with nature.	38	Intervention over 3 weeks. First week included 1×/day VR; second week, no intervention; third week included VR every day.	INS; EQ-5D-5L; Brief Pain Inventory Short Form; Functional Assessment of Chronic Illness Therapy—Fatigue; Depression, Anxiety, and Stress Scale; Functional Assessment of Chronic Illness Therapy—Spiritual Well-being.	Statistically less fatigue, less depression, greater QOL and spirituality	Breast
Emami, E. et al. (2018). [28] Iran	Descriptive correlational	Examine the effect of nature on positive distraction on the health process of patients with cancer.	80	Views of natural landscapes compared with views of no natural landscape.	STAI, VAS pain (0–10).	Admitted patients viewing natural scenery had statistically significant less anxiety and pain than admitted patients viewing no natural scenes.	Stage 1 (21), 2 (25), 3 (27), 4 (7)
Kim, H. et al. (2019). [29] Korea	Exploratory study	What is the effect of forest therapy on the quality of sleep in patients with cancer?	9	6 days of forest therapy, 30 min of forest healing recreation and 40 min of meditation each day.	PSG, STOP BANG, Pittsburgh Sleep Quality Index, Stanford and Epworth Sleepiness, Hospital Anxiety and Depression Scale.	Sleep efficiency and total sleep time increased.	GI
Morris, S. L. et al. (2021). [14] Canada	Quasi- experimental, mixed methods	Determine if incorporating One Nature Challenge (ONC) offers additional psychological and/or physiological benefits.	18	A ten-week group-exercise program for individuals living with cancer.	Short Form Health Survey, Functional Assessment of Chronic Illness Therapy—Fatigue, Brief Fatigue Inventory, Patient-Specific Functional Scale, Orientation to Life Questionnaire, Seniors Fitness Test, hand grip strength, VS.	No gain in overall health was found between groups. Aerobic fitness and fatigue improved for the ONC group.	Not reported
Nakau, M. et al. (2013). [30] Japan	Pilot quantitative interventional	Examine the effect of spiritual care of cancer patients by integrated medicine in a green environment.	22	Forest therapy, horticultural therapy, yoga meditation, support group therapy 1×/week ×12 weeks	Functional Assessment of Chronic Illness Therapy—Spiritual Well-being, Short Form-36 Health Survey Questionnaire, Cancer Fatigue Scale, POMS, STAI, natural killer cell activity.	Significant improvements in functional well-being and spiritual well-being, QoL cancer-related fatigue, tension/anxiety and confusion were reduced, NK cell activity significantly increased.	Breast and lung
Pearson, A. L. et al. (2021). [13] United States	Cross-sectional survey	Evaluate the change in active and passive use of nature, places of engaging with nature, and association of nature contact with respect to improvements to perceived stress and symptom experience among breast cancer patients during the pandemic.	56	Survey	Investigator-developed tool regarding nature interactions, Nature-Relatedness 6, PSS, MD Anderson Symptom Inventory.	Decreased use of parks was significantly related to higher stress; increased usage of backyard/porch was significantly associated with lower stress.	Breast
Scates, D. et al. (2020). [12] United States	Repeated-measure experimental design	The purpose of this research was to determine whether a nature-inspired VR simulation reduced stress and pain levels among patients in a cancer treatment center.	50	VR during IV or port procedure, control was usual IV or port procedure.	Demographic questions, 8 investigator-developed questions on stress and pain.	Patients felt more relaxed and distracted during VR.	N/A
Victorson, D. (2021). [31] United States	Single-arm within- subjects program evaluation	Examine outcomes following participation in immersive, multi-night, mindfulness-based treks in nature in a sample of young adults and caregivers.	50	Week-long backpack country trek or long-weekend trek at a retreat center.	C-reactive protein (CRP) and Interleukin-6 (IL-6), connection, knowledge, efficacy, enjoyment, appreciation, insights, and learning, PROMIS Anxiety, Depression and Sleep Disturbance short forms.	Improvements in feeling connected to nature, peers, and oneself; improvements in anxiety, depression, and sleep disturbance; and changes in proinflammatory biomarkers.	Breast, blood, brain

**Table 3:** Measures of variables.

Variable	Measures
Anxiety	State-Trait Anxiety Inventory (STAI) [28,30] Depression, Anxiety, and Stress Scale Short Form (DASS-21) [11] Hospital Anxiety and Depression Scale [29] PROMIS Anxiety Short Form (4A) [31]
Stress	Depression, Anxiety, and Stress Scale Short Form (DASS-21) [11] Perceived Stress Scale (PSS) [13] Investigator developed [12]
Depression	Depression, Anxiety, and Stress Scale Short Form (DASS-21) [11] Hospital Anxiety and Depression Scale [29] PROMIS Depression Short Form (4A) [31]
Pain	Brief Pain Inventory [11] Visual Analog Scale [28] Investigator developed [12]
Fatigue	Functional Assessment of Chronic Illness—Fatigue [11,14] Brief Fatigue Inventory [14] Cancer Fatigue Scale [30]
Sleep	Polysomnography (PSG), STOP BANG, Pittsburgh Sleep Quality Index, Stamford and Epworth Sleepiness Scales [29] PROMIS Sleep Disturbance Short Form (4A) [31]
Quality of life	EQ-5D-5L [11] Short Form-36 Health Survey Questionnaire [30]
Nature relatedness	Nature-Relatedness 6 [13,14]
Spiritual well-being	Functional Assessment of Chronic Illness Therapy—Spiritual Well-being [11,30]
Well-being	Investigator developed [16]
Cancer identity	Investigator developed [16]
Nature connectedness	Inclusion of Nature in the Self (INS) [11]
Symptom inventory	MD Anderson Symptom Inventory [13]
Psychological state	Profile of Mood States (POMS) [30]
Function	Patient-Specific Functional Scale [14]
Inflammatory biomarkers	C-Reactive Protein (CRP) and Interleukin-6 (IL-6) [31]

## Data Availability

Not applicable.
